# A National study of burdensome health care costs among non-elderly Americans

**DOI:** 10.1186/1472-6963-14-435

**Published:** 2014-09-25

**Authors:** Ilana B Richman, Mollyann Brodie

**Affiliations:** Center for Health Policy/Center for Primary Care and Outcomes Research, Stanford University School of Medicine, Stanford, CA USA; Public Opinion and Media Research, Kaiser Family Foundation, Menlo Park, CA USA

**Keywords:** Health care costs, Health insurance, Health economics

## Abstract

**Background:**

Rising health care costs and increased cost sharing have resulted in significant medical expenses for many Americans. The goal of this study was to describe the prevalence of and risk factors for burdensome health care costs among non-elderly Americans.

**Methods:**

This was a cross sectional study of a nationally representative sample of non-elderly Americans. We used survey data previously collected by the Kaiser Family Foundation. We used logistic regression to identify key risk factors for burdensome health care costs and to assess whether risk factors differ according to age within our study population. For analyses comparing younger and middle-aged adults, we compared participants ages 18–39 (younger Americans) to those ages 40–64 (middle-aged Americans).

**Results:**

Our study population included 5,493 participants. Twenty seven percent of participants reported difficulty paying medical bills, a prevalence that did not differ by age. Low income, lack of health insurance, and poor health were independently associated with difficulty paying medical bills after controlling for demographic covariates. Both younger and middle-aged adults were likely to experience burdensome health care costs at low incomes. At moderate incomes, risk fell for middle-aged adults, but remained high for younger adults (OR_middle-age_ 1.40, 95% CI 1.12-1.75, OR_younger_ 2.48, 95% CI 1.73-3.57, p value for interaction 0.004). Younger adults without insurance were at risk for accruing burdensome costs compared to their insured counterparts (OR 2.61, 95% CI 1.96-3.47). Middle-aged adults without insurance, though, had an even higher risk (OR 3.82, 95% CI 2.93-4.97, p value for interaction 0.037).

**Conclusions:**

Both younger and middle-aged adults commonly report difficulty paying medical bills. Younger adults remain vulnerable to burdensome medical costs even when earning moderate incomes. Middle-aged adults, however, are more likely to encounter burdensome costs when uninsured. These findings suggest that younger and middle-aged adults experience distinct vulnerabilities and may benefit differentially from health reform efforts intended to expand coverage and limit out-of-pocket expenses.

## Background

In recent years, rising health care costs and increased cost sharing have resulted in significant medical expenses for many Americans [1]. Vulnerable populations, including the poor and uninsured, are especially likely to have difficulty paying for medical care, though many middle class Americans also struggle to pay medical bills [1–3]. Americans who have difficulty paying for medical care frequently report avoiding care in an effort to limit costs [4]. High out-of-pocket costs for medical services may translate not only into lower rates of utilization but also worse health outcomes for the poorest and sickest patients [5]. Medical bills can also have serious financial consequences, resulting in depletion of savings or even bankruptcy [6, 7].

Nonelderly adults (those ages 18–64) experience fewer burdensome costs compared to those 65 and older, though they are by no means immune [3, 8]. Nonelderly adults represent a diverse population and within the group, varying financial, health, and demographic factors may contribute to burdensome medical costs. Middle-aged adults have more chronic medical conditions compared to younger adults, though many younger adults also face health problems that require ongoing medical care [9–11]. Younger adults are more likely to be injured by accidents, violence, and by self-harm compared to middle-aged adults [9, 12]. Younger adults also have unique financial constraints with lower incomes and lower rates of insurance compared to middle-aged, working adults [13, 14]. Whether younger and middle-aged adults experience different financial burdens related to health care costs has not been well described.

This study examined the financial burdens of health care among the non-elderly population in general, and on the younger and middle-aged adults within that group. First, we defined the prevalence of burdensome medical costs among all non-elderly Americans and among younger and middle-aged adults within this group. We identified risk factors for burdensome costs in the non-elderly population as a whole. We then asked whether these risk factors differentially affected ability to afford medical care among younger adults compared to middle-aged adults within this group. We hypothesized that younger and middle-aged adults might be susceptible to similar risk factors for burdensome health care costs but that these risk factors may affect younger and middle-aged adults differently.

## Methods

This was a cross sectional study of a representative sample of American adults. Data for this study were collected previously through Kaiser Family Foundation (KFF) health tracking polls. KFF health tracking polls are monthly telephone surveys of representative samples of Americans. Surveys are designed to assess experience with and opinions about our health care system. This study aggregated data from the KFF health tracking polls conducted in March 2010, June 2010, December 2010, March 2011, August 2011, and May 2012, the six most recent polls that assessed burdensome health care costs. For each monthly poll, participants were selected randomly from among Americans with access to a landline or cell phone using a random digit dialing procedure. There was no oversampling of specific populations. Each survey wave was conducted independently and participants were not tracked longitudinally over time. All data were self-reported. Surveys were carried out in English or Spanish by Princeton Research Associates.

Respondents were adults age 18 and older who agreed to participate and spoke English or Spanish. For the purposes of this study, we excluded any participant age 65 or older. Most of those respondents are covered by Medicare and, as a group, experience markedly different costs compared to the population under age 65 [15]. We did include adults below the age of 65 covered by Medicare. These participants are likely eligible for Medicare due to disability or end stage renal disease and excluding them would have selected for an overall healthier study population.

Our primary outcome variable was participant-reported burdensome medical costs. To assess burdensome medical costs, we asked all participants the following question: “In the past 12 months, did you or another family member in your household have any problems paying medical bills or not?” We evaluated income, health insurance status, and health insurance type as predictors of burdensome medical costs. We also examined self-reported health status, rated from “excellent” to “poor”, as predictors. Basic demographics such as age, sex, ethnicity and race were included in analyses as covariates. For participants who did not report age, many selected an age category that described their age. For these participants (n = 81), age was estimated as the median age in the reported group.

We performed statistical analyses using Stata version 11.0. For all analyses, we weighted participants on age, sex, race, ethnicity, region, population density, education, and household phone use. Hispanic participants in the May 2012 poll were also weighted on nativity. Weights were designed to allow our study population to most closely reflect the demographics of the US population as a whole. Weights for demographic variables were derived using population estimates from the US Census Bureau’s Annual Social and Economic Supplement Survey. Population density weights were derived from the US Decennial Census. Household phone use weights used data from the National Health Interview Survey. Weights also accounted for the fact that participants with both a cell phone and landline were more likely to be contacted.

For our bivariate analyses, we divided our sample at age 40 to compare younger versus middle-aged adults. We chose this cut point because adults 18–39 have health concerns distinct from those 40–65. Younger adults have fewer chronic conditions including heart disease, cancer, and diabetes compared to middle-aged adults, but are more likely to suffer from catastrophic illness such as trauma [10–12]. Descriptive analyses used weighted means, medians and proportions. For bivariate analyses, we compared weighted proportions using the Wald test. Multivariable models used logistic regression and were run in Stata’s “svy” mode to account for weights. We ran two sets of logistic regression analyses. The first set of models was intended to identify predictors of burdensome health care costs among our study population as a whole. We first modeled each predictor we hypothesized to be associated with the outcome in an unadjusted model to quantify any apparent association between each predictor and the outcome. We then created a joint model in which we included each of these predictors. We also added other demographic variables such as race, ethnicity, and sex, based on face validity.

In our second model, we explored whether the magnitude of association between these predictors and the outcome, burdensome medical costs, varied according to the age of the participant. To evaluate this quantitatively, we created a model that included these primary predictors and an interaction term representing an interaction between the predictor and age as a continuous variable. Interaction terms were retained in the final model if the p-value for interaction was <0.1. We also again included demographic covariates including sex, race, and ethnicity in the final model. Finally, we used linear combinations of odds ratios at representative ages (the mean ages in the 18–39 and 40–64 groups) to demonstrate how the odds of experiencing burdensome costs differ among younger and middle-aged adults.

Logistic regression models were checked for model fit using the Hosmer-Lemeshow test and were evaluated for influential points using delta-beta statistics. These post estimations were done in unweighted models which closely resembled weighted models.

This study was exempt from review by the Stanford University Institutional Review Board.

## Results

Our initial sample included 7,243 participants. The six Kaiser Family Foundation health tracking polls used in this study had response rates that ranged from 18-24%, rates typical for public opinion polls [16]. After excluding adults age 65 and older (n = 1,703) and participants with missing data (n = 47), our sample included 5,493 participants. In order to characterize demographic differences among the younger and middle-aged groups, we divided our sample into adults ages 40–64 (referred to hereafter as “middle-aged adults”, n = 3,496) and those 18–39 (referred to hereafter as “younger adults”, n = 1,997). General demographic characteristics of the population are detailed in Table 1. Overall, the study population was 50% female with no significant sex difference among middle-aged and younger adults. The mean age for the study population was 41, with a mean age of 29 among the younger group and 51 among the middle-aged group. The younger group had a larger proportion of African American participants (16% vs. 12%, p < 0.001) and Hispanic participants (21% vs. 11%, p < 0.001). Younger adults tended to have lower incomes. A full third of younger adults had incomes below $30,000 a year, compared to 26% of middle-aged adults (p < 0.001). Younger adults also more commonly relied on Medicaid for health insurance (7.6% vs. 4.1%, p < 0.001) and were more likely to be uninsured (24% vs. 18%, p < 0.001) compared to middle-aged adults. Middle-aged adults, by contrast, were more likely to report being in fair or poor health than younger adults (20% vs. 11%, p < 0.001).

**Table 1:** **Demographic characteristics**

Variable	Overall* (N = 5493)	Age 18–39 (N = 1997)	Age 40–64 (N = 3496)	p-value
Difficulty paying for health care	27%	27%	28%	0.86
Mean age (SE)	41 (0.21)	29 (0.16)	51 (0.13)	<0.001
Sex (male)	50%	51%	49%	0.30
Income (dollars per year)				
<30,000	29%	33%	26%	<0.001
30,000-50,000	37%	37%	37%	0.54
>50,000	24%	20%	28%	<0.001
DK/NR	10%	10%	10%	0.86
Race				
White	74%	69%	79%	<0.001
Black/African Am.	14%	16%	12%	<0.001
Asian	3.5%	5.4%	1.9%	<0.001
Mixed/other	5.7%	7.4%	4.2%	<0.001
DK/NR	2.6%	2.3%	2.8%	0.28
Ethnicity				
Hispanic	15%	21%	11%	<0.001
Non Hispanic	84%	79%	89%	<0.001
DK/NR	0.6%	0.4%	0.7%	0.33
Uninsured	21%	24%	18%	<0.001
Health insurance type				
Employer based	65%	59%	69%	<0.001
Self insured	13%	12%	13%	0.46
Medicare	6.5%	5.0%	7.7%	0.004
Medicaid	5.6%	7.6%	4.1%	<0.001
Other government	4.7%	4.8%	4.6%	0.81
Other (including parents’ insurance)	5.1%	11%	1.0%	<0.001
DK/NR	0.5%	0.5%	0.4%	0.92
Health status				
Excellent, very good or good	84%	89%	80%	<0.001
Only fair or poor	16%	11%	20%	<0.001
DK/NR	0.04%	0.02%	0.02%	0.18

*Totals in each column may not sum to 100 due to rounding.

Despite a number of demographic differences, both younger and middle-aged adults reported similar rates of difficulty paying for health care. About 27% of respondents in each group reported having trouble paying a medical bill in the past year. Subsequent analyses focused on describing the factors associated with burdensome health care costs in the non-elderly population as a whole, and elucidating whether these factors contributed differentially to burdensome health care costs according to age.

We used multivariable logistic regression to identify factors associated burdensome costs in our study population (Table 2). In initial models, we included a single primary predictor of interest. These unadjusted models identified insurance status, health status, and income as factors most strongly associated with difficulty paying for medical expenses. We then combined these predictors in a single model to ask whether each was independently associated with difficulty affording health care. We also adjusted for age, sex, race, and ethnicity. In this combined model, those in fair or poor health had were more likely to have trouble paying for health care compared to those in good or excellent health (OR 2.60, p < 0.001). Income was also predictive of difficulty paying for medical care. Those who earned less than $30,000 a year were more likely to have trouble paying for health care than those who made more than $50,000 a year (OR 2.30, p < 0.001). Insurance status was strongly associated with difficulty paying for medical care. Those without insurance were much more likely than those with employer sponsored insurance to have trouble paying for health care (OR 3.11, p < 0.001) and participants with Medicare were more likely to report burdensome medical costs (OR 1.43, p = 0.04). Lastly, women were somewhat more likely to report difficulty paying for medical care compared to men (OR 1.41, p < 0.001).

**Table 2:** **Predictors of burdensome medical costs**

Unadjusted models	Predictor	OR (95% CI)	p-value
Model 1: Age	Age (continuous)	1.00 (0.99-1.00)	0.48
Model 2: Health Status	Excellent, very good, or good (Ref.)	--	--
	Fair or poor	3.75 (3.13-4.50)	<0.001
Model 3: Income	Income >50 K (Ref.)	--	--
	Income 30-50 K	2.18 (1.78-2.67)	<0.001
	Income <30 K	4.94 (4.02-6.09)	<0.001
Model 4: Insurance	Employer (Ref.)	--	--
	Self pay	0.84 (0.65-1.10)	0.22
	Medicare	2.71 (1.99-3.69)	<0.001
	Medicaid	2.55 (1.80-3.62)	<0.001
	Other government insurance	1.36 (0.91-2.03)	0.11
	Other, including parents’ insurance	0.83 (0.53-1.31)	0.42
	No insurance	4.66 (3.89-5.58)	<0.001
**Combined model**	**Predictor**	**OR (95% CI)**	**p-value**
Model 5: health status + income + insurance + ethnicity + race + age + sex*	Poor health	2.60 (2.12-3.20)	<0.001
	Income 30-50 K	1.73 (1.40-2.13)	<0.001
	Income <30 K	2.30 (1.79-2.95)	<0.001
	Self pay insurance	0.82 (0.62-1.07)	0.15
	Medicare	1.43 (1.02-2.03)	0.04
	Medicaid	1.32 (0.88-2.00)	0.18
	Other government insurance	1.01 (0.66-1.54)	0.99
	Other, including parents’ insurance	0.86 (0.54-1.38)	0.53
	No insurance	3.11 (2.53-3.83)	<0.001
	Hispanic	1.03 (0.80-1.32)	0.82
	African American	1.23 (0.98-1.51)	0.063
	Asian	0.76 (0.44-1.32)	0.33
	Age	1.00 (0.99-1.01)	0.82
	Female sex	1.41 (1.21-1.64)	<0.001

*Reference groups for Model 5 same as in Models 2–4, reference group for ethnicity and race is white/Caucasian group.

Our final set of analyses focused on whether the risk factors identified in our population as a whole differentially affected middle-aged and younger adults. We modeled the three strongest predictors of burdensome health care costs (income, insurance, and health status) with predictor-age interaction terms in a single model. We also again adjusted for sex, ethnicity, and race. To interpret the analyses, we evaluated the models at ages 29 and 51, the mean ages in among the middle-aged and younger age groups in our population. Table 3 details the results from these analyses. Among all participants, income of less than $30,000 a year was associated with an increased risk for burdensome medical costs. For younger adults in the next income range, though, the risk of burdensome medical costs remained high, while for middle-aged adults, it fell. For a 29 year old earning $30-50,000/year, the odds ratio for burdensome health care was 2.48 (p < 0.001) compared to those earning more than $50,000/year. For an adult of 51, the odds ratio at the same income was 1.40 (p < 0.001, p-value for interaction 0.004).

**Table 3:** **Interactions bsetween age and risk factors for burdensome costs**

Predictor	Age 29* OR (95% CI)	p-value	Age 51* OR (95% CI)	p-value	p-value for interaction
Income (dollars per year)					
>50,000 (Ref.)	--	--	--	--	--
30,000-50,000	2.48 (1.73-3.57)	<0.001	1.40 (1.21-1.75)	<0.001	0.004
<30,000	2.87 (1.94-4.25)	<0.001	2.07 (1.55-2.77)	<0.001	0.140
Insurance					
Employer based (Ref.)	--	--	--	--	--
Uninsured	2.61 (1.96-3.47)	<0.001	3.82 (2.93-4.97)	<0.001	0.037
Health status					
Excellent or Good (Ref.)	--	--	--	--	--
Poor or Fair	2.31 (1.64-3.28)	<0.001	2.90 (2.33-3.59)	<0.001	0.22

*Ages 29 and 51 were the mean ages in the 18–39 and 40–64 age groups. These ages were chosen as representative values for assessing any interaction between age and risk factors for burdensome medical costs.

Among middle-aged adults, by contrast, lack of health insurance was associated with greater risk of medical insecurity than among younger adults. An uninsured 51 year old had a near four-fold increased odds of experiencing burdensome costs compared to a peer with employer sponsored insurance (OR 3.82, p < 0.001). The odds ratio associated with being uninsured at the age of 29, by comparison, was 2.61 (p < 0.001, p-value for interaction 0.037).

Poor health put both young and middle-aged adults at risk for medical insecurity. For a 51 year old in poor or fair health, the odds of experiencing burdensome health care costs were 2.90 times that of their healthier peers. For a 29 year old, the odds ratio for burdensome health care costs associated with poor health was 2.31 (p < 0.001). The p-value for interaction in this model, though, did not reach statistical significance (p = 0.20).

## Discussion

Burdensome health care costs are common in the United States. Our study has shown that within the non-elderly population, over a quarter of adults have experienced difficulty paying for medical care. Lower incomes, lack of insurance, and poor health all contribute to difficulty paying for health care, findings consistent with previous studies of burdensome costs [3, 4]. Interestingly, we also found that difficulty paying for health care cut across demographic groups, with as many younger adults reporting trouble paying medical bills as middle-aged adults. This finding is somewhat surprising given that younger and middle-aged adults experience quite different financial pressures and different health challenges.

We have explored whether low income, lack of health insurance, and poor health differentially affected middle-aged and younger adults in our study population. We found that indeed, middle-aged and younger adults experience different pressures and challenges when paying for medical care. Younger adults had lower incomes overall, an independent risk factor for burdensome health care costs. Not only were younger adults poorer, but even at moderate incomes, younger adults were less able to pay for care compared to their middle-aged counterparts. This finding held after controlling for health insurance and health status. These results suggest that at moderate incomes, and even while insured, younger adults remain vulnerable to accruing burdensome costs. Younger adults may have fewer savings or more expenses, making medical bills more difficult to pay. Furthermore, insurance plans held by this population may not be sufficient to protect younger adults from burdensome costs. Income, savings and other resources may also matter when faced with medical bills.

Middle-aged adults also had unique vulnerabilities. Middle-aged adults without health insurance were at greater risk of experiencing burdensome costs compared to younger adults without insurance. This added risk with middle-aged age may be because middle-aged adults are more likely to fall ill during any given period without insurance, or are more likely to have a costly and debilitating illness during a period without insurance. Younger adults who fall ill and who have gaps in insurance are still at risk for burdensome medical expenses but perhaps are protected by the physical resilience associated with younger age. We saw a suggestion of an age-health status interaction that would also support the notion that when ill, middle-aged adults are more vulnerable than younger adults, though this interaction did not reach statistical significance.

This study has a number of limitations. First, all data are cross sectional, and though we have found associations between income, insurance, health status, and burdensome costs, we cannot assign causality. Indeed, it is possible, for example, that burdensome medical costs might lead to lower utilization of health care services and poor health, rather than poor health and high utilization leading to burdensome medical costs. Still, it seems plausible that scarce resources and poor health might result in high rates of costly medical expenses.

Second, our main outcome measure had some limitations. The question we used to assess burdensome medical costs asked not only about an individual’s difficulty paying medical bills, but whether anyone in the household had had difficulty paying medical bills. Respondents who live with family members of a different age who have had trouble paying medical bills might respond on their behalf. This limitation, though, would tend to bias our results toward the null rather than toward a positive finding relating to age. The question is also limited by its subjective nature. We did not ask about specific outcomes such as bankruptcy, but rather “difficulty” paying medical bills, which is open to interpretation and may capture people who simply found paying medical bills unpleasant rather than truly burdensome. In other studies of medical costs, though, this question has been found to correlate well with those who pay >10% of their income toward medical costs, a more objective measure of burden [1].

Lastly, our analyses were primarily exploratory. Our analyses were aimed at identifying risk factors and interactions. Exploratory analyses in general, and analyses that assess for interactions in particular, can produce false positive results. Importantly, though, our first finding that lack of insurance, low income, and poor health are risk factors for burdensome costs are supported by prior studies, as is our overall estimate of the prevalence of burdensome costs [4]. The interactions we explored here have not previously been reported. There are, however, plausible interpretations for these findings, making their validity more likely. We also explored interactions only among our strongest predictors in an effort to limit false positive results.

## Conclusions

In summary, our study has shown that over a quarter of non-elderly Americans experience trouble paying for medical care and that both younger and middle-aged Americans within that group share in this difficulty equally. Younger and middle-aged Americans, though, have different vulnerabilities, with younger Americans more sensitive to income pressures and middle-aged Americans more susceptible to high health care costs when they do not have insurance. These differences are important in guiding policies that target and address the specific needs of various populations. The Patient Protection and Affordable Care Act (ACA) includes a number of provisions which may help buffer against burdensome costs. Indeed, the ACA expands coverage to previously uninsured populations, limits yearly out-of-pocket expenses, and eliminates lifetime caps on essential benefits for beneficiaries. Future analyses examining burdensome costs after full implementation of the ACA may reveal the ways in which the law has helped protect Americans or whether the population-specific vulnerabilities identified in this study require additional attention.

## Authors’ information

Mollyann Brodie is the Director of Public Opinion Polling and Research at the Kaiser Family Foundation and is responsible for all aspects of the Foundation’s public opinion survey efforts, including the monthly Kaiser Health Tracking poll. The Kaiser Family Foundation is a non-partisan, non-profit foundation that provides information and analysis on topics in health care in the US. Ilana Richman is a fellow in Health Services Research at Stanford University and the Palo Alto Veterans Affairs Hospital.
